# A patient survey of the impact of fibromyalgia and the journey to diagnosis

**DOI:** 10.1186/1472-6963-10-102

**Published:** 2010-04-26

**Authors:** Ernest Choy, Serge Perrot, Teresa Leon, Joan Kaplan, Danielle Petersel, Anna Ginovker, Erich Kramer

**Affiliations:** 1 Sir Alfred Baring Garrod Clinical Trials Unit, Academic Department of Rheumatology, King's College London, UK; 2 Service de Médecine Interne et Centre de la Douleur, Hôtel-Dieu, Paris, France; 3 Pfizer Inc, 235 East 42nd Street, New York, NY 10017, USA; 4 Harris Interactive, Independence Way, Princeton, New Jersey, USA

## Abstract

**Background:**

Fibromyalgia is a painful, debilitating illness with a prevalence of 0.5-5.0% that affects women more than men. It has been shown that the diagnosis of fibromyalgia is associated with improved patient satisfaction and reduced healthcare utilization. This survey examined the patient journey to having their condition diagnosed and studied the impact of the condition on their life.

**Methods:**

A questionnaire survey of 800 patients with fibromyalgia and 1622 physicians in 6 European countries, Mexico and South Korea. Patients were recruited via their physician.

**Results:**

Over half the patients (61%) were aged 36-59 years, 84% were women, and the mean time since experiencing fibromyalgia symptoms was 6.5 years. Patients had experienced multiple fibromyalgia symptoms (mean of 7.3 out of 14), with pain, fatigue, sleeping problems and concentration difficulties being the most commonly reported. Most patients rated their chronic widespread pain as moderate or severe and fibromyalgia symptoms were on average "fairly" to "very" disruptive, and had a "moderate" to "strong" impact on patients' lives. 22% were unable to work and 25% were not able to work all the time because of their fibromyalgia. Patients waited on average almost a year after experiencing symptoms before presenting to a physician, and it took an average of 2.3 years and presenting to 3.7 different physicians before receiving a diagnosis of fibromyalgia. Patients rated receiving a diagnosis as somewhat difficult on average and had difficulties communicating their symptoms to the physician. Over one third (35%) felt their chronic widespread pain was not well managed by their current treatment.

**Conclusions:**

This survey provides further evidence that fibromyalgia is characterized by multiple symptoms and has a notable impact on quality of life and function. The diagnosis of fibromyalgia is delayed. Patients wait a significant period of time before presenting to a physician, adding to the prolonged time to diagnosis. Patients typically present with a multitude of symptoms, all resulting in a delay in diagnosis and eventual management. Helping clinicians to diagnose and manage patients with fibromyalgia should benefit both patients and funders of healthcare.

## Background

Fibromyalgia is a chronic, painful condition, with worldwide prevalence estimates ranging between 0.5% and 5.0% [1], that affects women approximately 7 times more often than men [2]. According to the American College of Rheumatology (ACR) classification criteria, fibromyalgia is characterized by widespread pain in all four quadrants of the body for at least 3 months, with pain on digital palpation in at least 11 out of 18 tender points [3]. In addition to pain, patients also commonly report other symptoms such as problems sleeping, fatigue, stiffness, problems with concentration, headaches, migraine, paresthesias and irritable bowel or bladder [3,4]. Clinically relevant levels of anxiety or depression have been identified in approximately one-third of patients [2,5]. Patients with fibromyalgia have reduced quality of life and often marked disability [4,6]. People with fibromyalgia have been shown to have greater overall health status impairment than those with other chronic pain conditions such as rheumatoid arthritis, osteoporosis and osteoarthritis [7].

Although fibromyalgia is the most common chronic widespread pain condition, it is often underdiagnosed [8]. The diagnosis of fibromyalgia has been shown to increase patient satisfaction [5] and reduce healthcare utilization [9]. This international survey of fibromyalgia examines the experience of patients, the impact of the disease on quality of life, time taken from symptom onset to diagnosis, and explores their journey to diagnosis. The survey was conducted to provide descriptive data.

## Methods

This survey of patients diagnosed with fibromyalgia was conducted between February 25^th ^and April 17^th^, 2008 in eight countries; France, Germany, Italy, Mexico, The Netherlands, South Korea, Spain, and the United Kingdom (UK). Patients were identified by physicians who treated them for fibromyalgia; the recruiting physicians either participated in a companion survey or were sampled specifically for the purpose of recruiting fibromyalgia patients, but did not complete the physician survey. Physicians who recruited patients were identified using proprietary databases and physician directories. Patients were compensated for completing the survey. As this was a non-interventional survey, no ethical approval was required nor sought.

In total, 681 patients (85%) were surveyed via telephone interview using computer assisted telephone interviewing (CATI) technology. Face-to-face interviews using a pen and paper survey were conducted in South Korea in compliance with cultural norms (19 patients in Mexico were interviewed face-to-face using CATI due to respondents' preference). In South Korea, it is more considerate to conduct such an interview (>15 minutes long) face-to-face rather than by telephone. Further, face-to-face interview generates more trust and some comfort level between the respondent and interviewer which is particularly important in Asian cultures.

The English questionnaire was translated into all languages by an independent professional translation agency, and then all translations were validated by another independent translation agency. The local operators who administered the questionnaires also reviewed the translations before collecting the data. The questionnaire, which took approximately 15 minutes to complete, included questions about demographic information, symptoms, the impact of fibromyalgia on aspects of their life, treatment, and about interactions with healthcare professionals. Questions included lists of possible answers that were proctored and self-administered by the patient. The survey is included in additional file 1-Survey of patients with fibromyalgia. Specific details of questions and responses are provided in the results below. Data were processed and quality assured. A post-hoc statistical analysis comparing variables between those patients who reported being satisfied and not satisfied with treatment was conducted. P values were based on z-test of column proportions.

In addition, a survey of physicians in the eight countries was conducted, which included questions about physician behaviors and perceptions related to fibromyalgia diagnosis. In each country primary care physicians (PCPs), rheumatologists, neurologists, psychiatrists and pain specialists were surveyed.

## Results

### Patients

In total, 800 patients and 1622 physicians completed the survey (100 patients, 100-103 PCPs and 100-103 specialists from each country). All patients confirmed that a physician had diagnosed their fibromyalgia. The demographic characteristics were generally similar across the eight countries. Overall, 84% of patients were women and were distributed by age group as follows: 19% 18-35 years, 26% 36-44 years, 35% 45-59 years, 16% 60-74 years, and 4% ≥75 years. In the Korean sample, there was a higher percentage of men (23%) than in the other countries (range 12-17%). The mean time since first experiencing fibromyalgia symptoms was 6.5 years (range across countries 4.5 years in Mexico to 8.4 years in Spain).

### Symptoms

**Figure 1:**
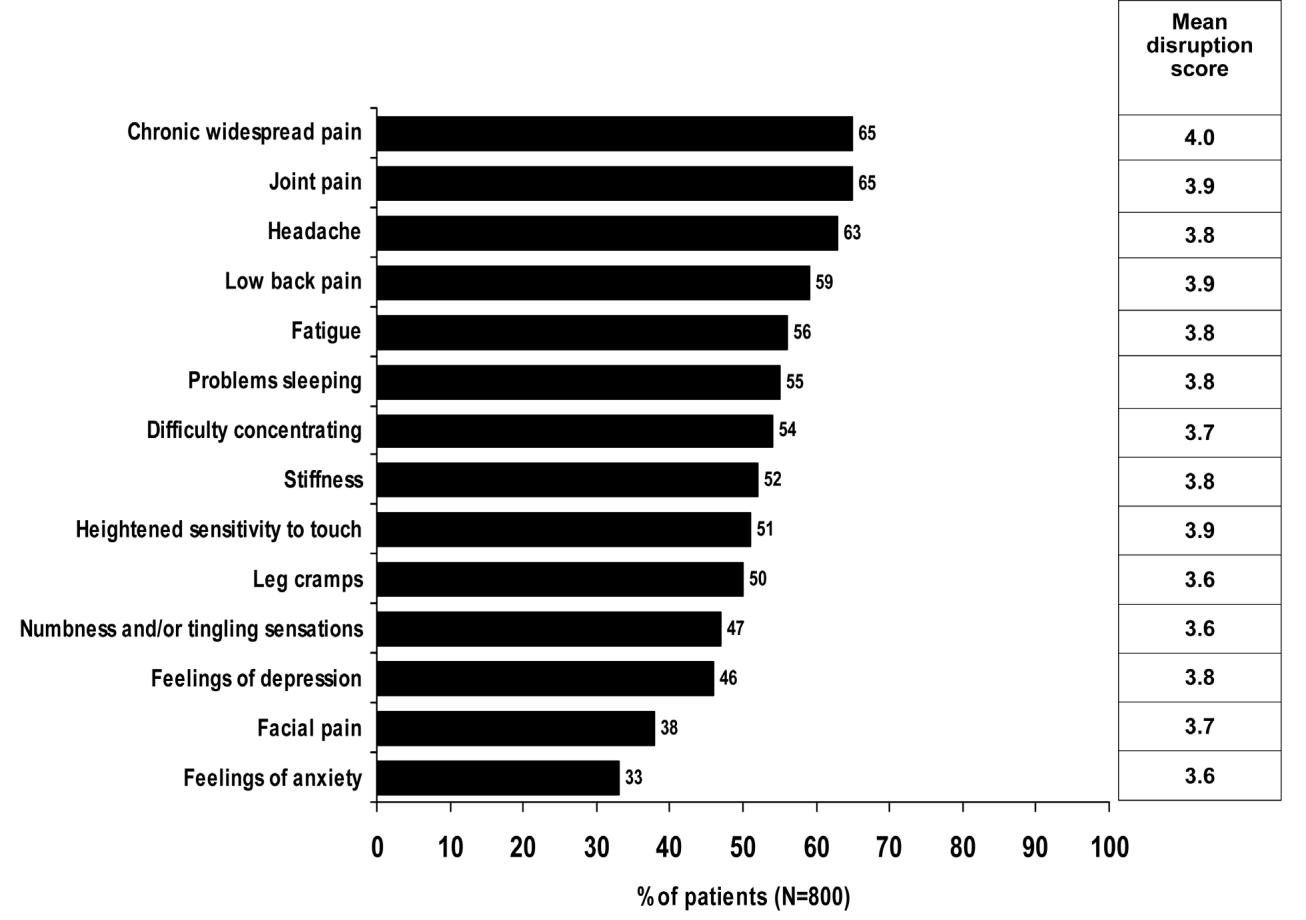
**Percentage of patients who reported experiencing each symptom listed and the mean disruption scores**. Disruption rated on a five-point scale from 1 = not at all disruptive, 2 = not very disruptive, 3 = fairly disruptive, 4 = very disruptive, 5 = extremely disruptive. Mean impact on QOL - higher score means greater impact.

The mean number of symptoms associated with fibromyalgia that patients had experienced at any time, from the list of 14 symptoms, was 7.3 and was similar across countries. All but two of the patients surveyed reported at least one pain symptom. Chronic widespread pain was reported by 65% of patients with other painful symptoms such as joint pain, headache and low back pain also being reported by more than half the patients (Figure 1). Non-painful symptoms including fatigue, sleeping problems, and concentration difficulties were also reported by over half the patients (Figure 1).

### Impact

Patients rated the extent of disruption to their overall quality of life from each of the symptoms they reported experiencing, on a five-point Likert scale from 1 = not at all disruptive to 5 = extremely disruptive. All symptoms were rated as being very or extremely disruptive by over half the patients who reported the symptom. Over 20% of patients rated chronic widespread pain, headaches, joint pain, low back pain, problems sleeping, stiffness, heightened sensitivity to touch and feelings of depression as extremely disruptive (5, maximum score). Mean disruption scores for each symptom were similar across countries. Across all symptoms overall mean disruption scores combined across countries ranged from 3.6 to 4.0 (Figure 1).

Patients who reported they had experienced chronic widespread pain were probed further about their pain. Overall, 17% said they experienced widespread pain every day, 10% 4 or 5 days per week, 38% 2 or 3 days per week, and 26% once per week; 8% responded less than once per week. Patients were asked to rate the severity of chronic widespread pain from fibromyalgia from 0 = no pain to 10 = worst possible pain. Chronic widespread pain was rated as severe (a score of 7 to 10) by 70% of patients and as moderate (a score of 4 to 6) by 28%.

**Figure 2:**
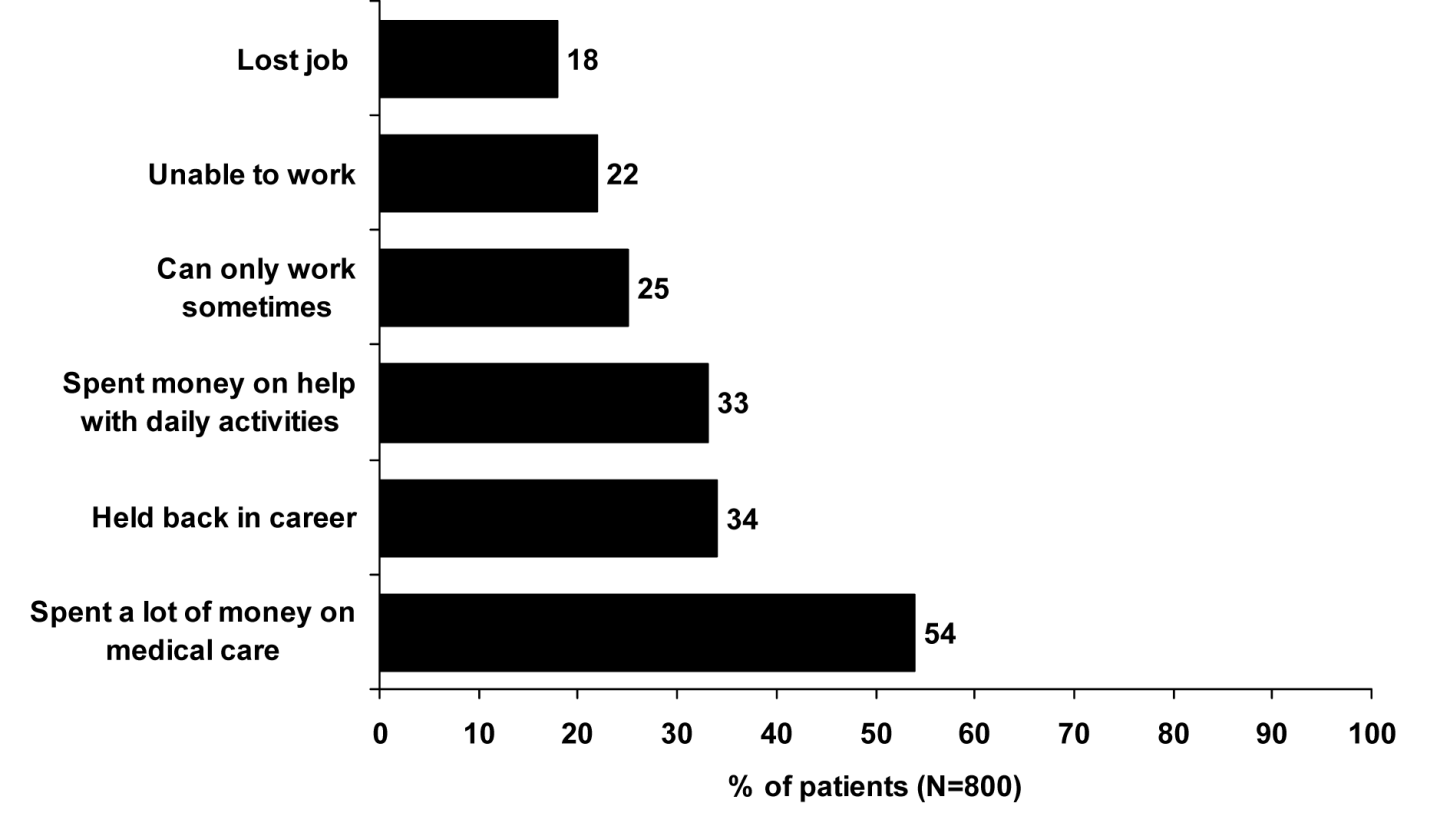
**Impact of fibromyalgia on employment and expenditure**. Patients were asked to rate the direct affect of fibromyalgia on employment and expenditure.

**Figure 3:**
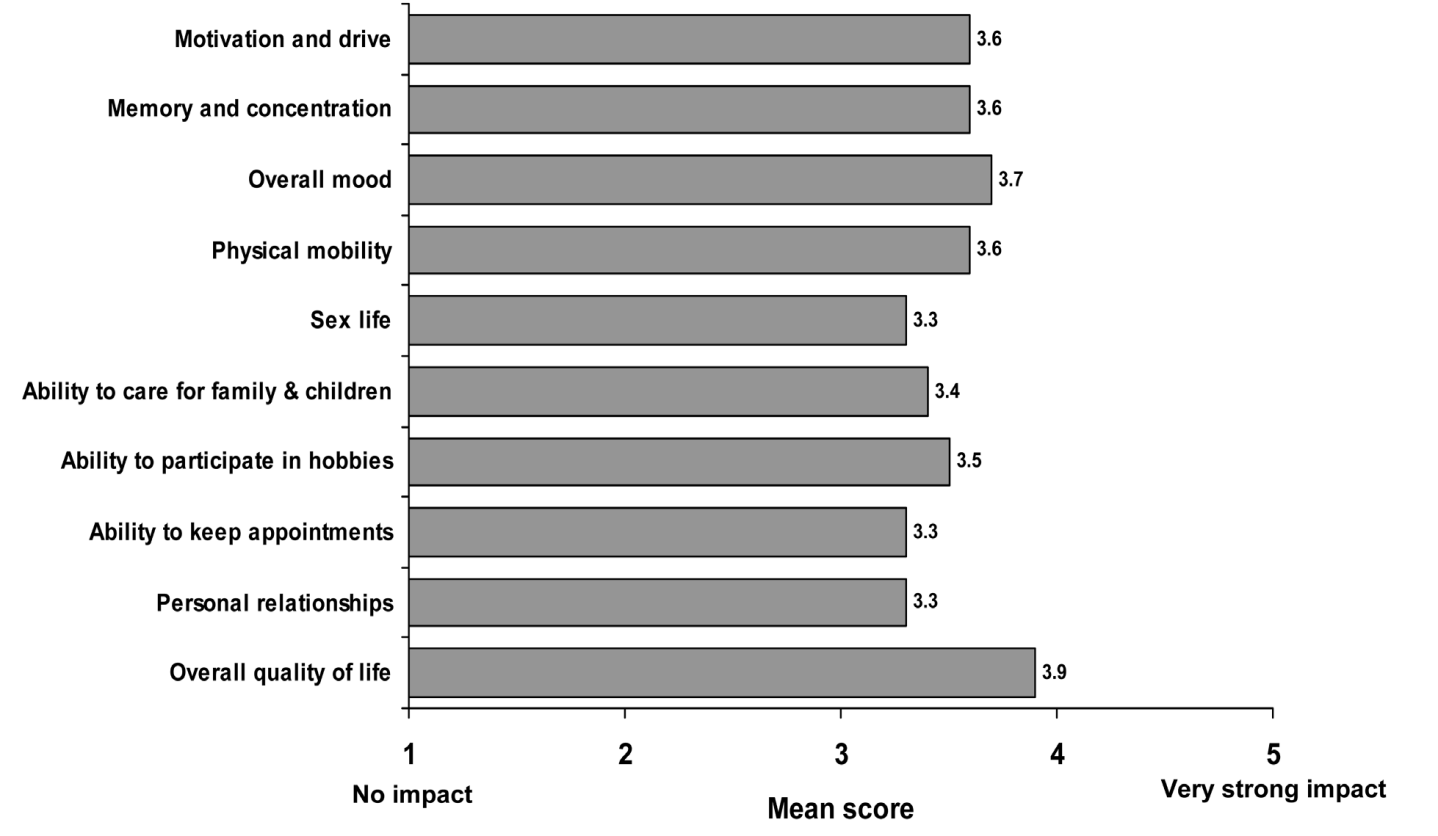
**Impact of fibromyalgia on aspects of life**. 1 = no impact, 2 = slight impact, 3 = moderate impact, 4 = strong impact, 5 = very strong impact.

Among those who had been employed for the last 12 months (n = 513), 48% had missed 10 or more working days in the last 12 months because of their fibromyalgia. A notable proportion of patients had their employment adversely affected by fibromyalgia at some time (Figure 2). On average, fibromyalgia had a moderate to strong impact on many aspects related to quality of life (Figure 3).

### Diagnosis of fibromyalgia

Patients were asked how long ago they first noticed symptoms of fibromyalgia and how long after noticing symptoms did they wait before presenting to a doctor. The mean time taken to present to a physician was 11.1 months (range across countries 4.6 months in the UK to 18 months in Mexico). In total, 29% of patients did not present to a physician for their symptoms until at least 6 months after the symptoms were first experienced. Patients were asked to respond to specific reasons as to why they waited to present to a doctor. The most common reasons were that patients thought the symptoms would resolve (74%), they could manage symptoms themselves (68%), they do not like receiving medications or injections (54%), the symptoms were not sufficiently severe (51%), and that they do not like going to the doctor (51%). Overall, 38% of patients gave one of the reasons for delaying presentation to a doctor as being afraid they would not be taken seriously, 44% were concerned about cost, 42% were too busy, and 29% found it difficult to schedule an appointment.

From the first time a patient presented to a physician about their fibromyalgia symptoms, the mean time to receiving an actual diagnosis of fibromyalgia was 2.3 years, with most countries being in the range of 2.1 to 2.7 years. The exceptions were Spain (3.7 years) and South Korea (0.6 years). The average number of physicians patients presented their symptoms to, before receiving a diagnosis of fibromyalgia was 3.7, with 38% of patients having presented to more than three physicians. Patients rated their overall difficulty in receiving a fibromyalgia diagnosis on a five-point Likert scale from 1 = very difficult to 5 = very easy, and the average score was 2.3, approximating to somewhat difficult. After the first consultation with a physician about their symptoms, patients in Europe most commonly presented to PCPs (95-98%), rheumatologists (62-72%), neurologists (51-61%), and psychiatrists (21-32%) about their fibromyalgia. In Europe the percentage of patients who presented to a pain specialist ranged from 14% in Italy to 46% in the Netherlands. Data on the types of physicians from Mexico and Korea were quite different from the European countries, due to quite different structures in their healthcare systems.

**Figure 4:**
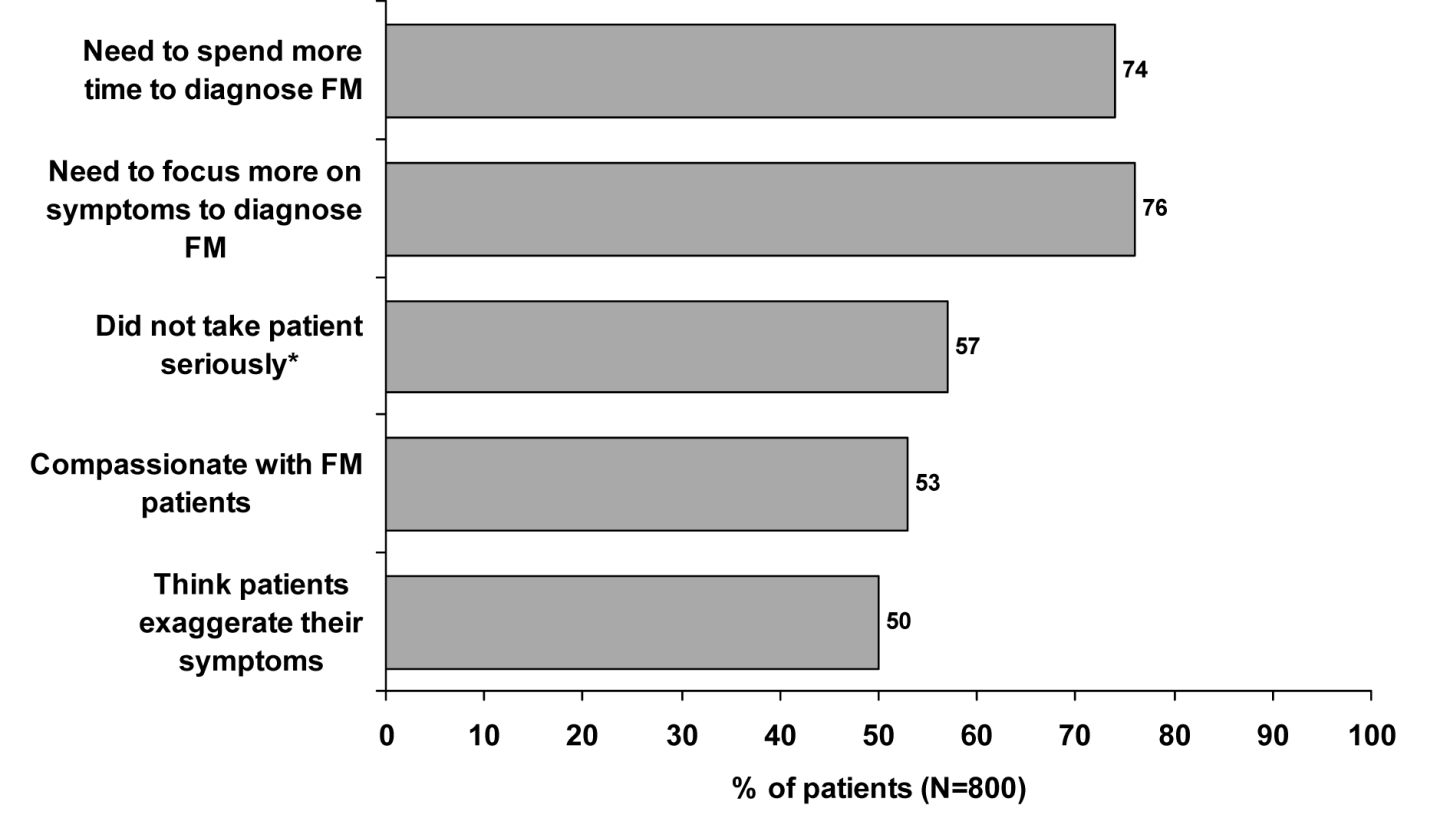
**Patients who somewhat or strongly agreed with statements about physicians during fibromyalgia diagnosis and management**. Patients rated statements about diagnosing and managing fibromyalgia 5 = strongly agree, 4 = somewhat agree, 3 = neither agree nor disagree, 2 = somewhat disagree, 1 = strongly disagree. * at least one experience.

At the time of the survey, patients were mostly being treated by PCPs, rheumatologists and neurologists (not mutually exclusive, and quite variable across countries). In total, 83% of patients were making fibromyalgia-specific visits to a physician at least once per month with 30% visiting a physician twice a month, and 11% visiting a physician three times a month. Overall, 59% of patients agreed that they had found it difficult to communicate their fibromyalgia to physicians. Patients' overall perceptions about some aspects of their experiences with physicians and fibromyalgia were captured. The percentages of patients who somewhat or strongly agreed with specific statements are shown in figure 4. Approximately three quarters of patients reported that doctors needed to spend more time and to focus more on symptoms to diagnose fibromyalgia.

In the companion survey of physicians, responses were obtained from 809 PCPs, 206 rheumatologists, 201 neurologists, 204 psychiatrists, and 202 pain specialists (n = 1622). Among these physicians, 24% strongly agreed and 40% somewhat agreed that it is difficult for patients to communicate symptoms of fibromyalgia to a physician. When asked how easy or difficult it was to diagnose fibromyalgia, 11% of physicians (12% of PCPs and 10% of specialists) responded that it was very difficult, and 42% of physicians (48% of PCPs and 35% of specialists) responded that it was somewhat difficult. Of the physicians surveyed, 45% (52% of PCPs and 39% of specialists) were not aware of the ACR fibromyalgia classification criteria [3]. Of those physicians who were aware of the ACR criteria and have diagnosed fibromyalgia patients in the past two years (n = 725), 26% did not use the ACR criteria when diagnosing fibromyalgia in their clinical practice.

### Treatment

Among the patients surveyed, 70% were using pain medications prescribed by the physician, 36% were using over the counter analgesics, 28% were using sleep aids and 56% were using other prescribed agents. Non-drug treatments were also commonly used with exercise (48%), relaxation techniques (45%), and lifestyle changes (44%) being the most frequently employed. Patients were asked to rate how satisfied they were with the ability of their current treatment to relieve fibromyalgia symptoms on a five-point Likert scale from 1 = not at all satisfied to 5 = extremely satisfied, with 3 representing fairly satisfied. The mean score was 2.9 and was similar across countries. Half of patients were fairly satisfied (50%) or very satisfied (19%), although 28% were not very satisfied or not at all satisfied. Chronic widespread pain was the most common symptom that patients reported as not well managed by their current treatment (35%), with fatigue, joint pain and concentration difficulties each not being well managed in 22% of patients.

### Relationship between treatment satisfaction, symptoms and treatment

Given that over one quarter of patients were not satisfied with their treatment, we explored some of the questions according to levels of treatment satisfaction to evaluate if there were any particular features that distinguished those who were satisfied from those who were not. Patients who were less satisfied with their treatment, on average experienced significantly more symptoms, rated their chronic widespread pain as being significantly more severe and disruptive and used significantly more treatments to manage their disease (Table 1). Compared to patients who were satisfied with their treatment, patients not satisfied with their treatment presented to significantly more physicians to receive a diagnosis and overall reported that receiving a diagnosis of fibromyalgia was more difficult (p < 0.05). In addition, the mean time to receiving a diagnosis was significantly longer in those who were less satisfied with their treatment (mean 3.1 years) compared to those who were satisfied (mean 1.4 years) (p < 0.05). Also, patients not satisfied with their treatment were more likely to miss more than 40 days of work, and/or more likely to claim that they have not been able to work or can only work some time, and/or more likely to lose their job due to fibromyalgia (p < 0.05).

**Table 1:** Fibromyalgia characteristics and treatment utilization among those satisfied and not satisfied with their current treatment

	Group A	Group B	
	**Not satisfied** **Patients with satisfaction score 1 or 2 (n = 223)**	**Satisfied** **Patients with satisfaction score 4 or 5 (n = 168)**	**P Value**

Mean time since first experiencing symptoms	7.3 years	5.5 years	<0.05

% of patients taking prescription pain medications	72%	60%	<0.05

% of patients taking OTC pain medicines	38%	33%	NS

% of patients taking other drugs prescribed by physician	52%	54%	NS

Average number of symptoms experienced	8.4	6.4	<0.05

Chronic widespread pain extremely or very disruptive	87%	59%	<0.05

Severity of chronic widespread pain	7.6	6.8	<0.05

Time lag between symptoms and seeing a physician	16.2 months	9.8 months	NS

Time lag between seeing a physician and receiving a diagnosis	3.1 years	1.4 years	<0.05

Average number of physicians seen to receive diagnosis	4.1	3.2	<0.05

Perceived difficulty in receiving diagnosis very or somewhat difficult	72%	47%	<0.05

Missed more than 40 days of work	19%	10%	<0.05

Unable to work due to fibromyalgia	36%	16%	<0.05

Can only work some time due to fibromyalgia	35%	21%	<0.05

Lost job due to fibromyalgia	30%	15%	<0.05

Average number of treatments used	3.9	3.4	<0.05

1. Not at all satisfied 2. Not very satisfied, 3. Fairly satisfied, 4. Very satisfied, 5 Extremely satisfied; p-value based on T-Test of Column Proportions

## Discussion

The results of this survey are from 800 patients and over 1600 physicians from eight different countries. The patient demographics were consistent with fibromyalgia patients in the general population. Although chronic widespread pain was the dominant symptom, fibromyalgia patients also experienced multiple symptoms (mean of 7.3 from a list of 14) in addition to pain. The most commonly reported symptoms were fatigue, problems sleeping and concentration difficulties, a finding which is consistent with other studies [6,10-12]. This survey of patients also confirms the negative impact of fibromyalgia which was reflected in average scores on particular aspects of life and function such as motivation, concentration, mobility, personal relationship and hobbies. The economic impact of fibromyalgia on employment was also notable, with almost half missing at least 2 weeks work in the last year because of their fibromyalgia, 22% actually unable to work at all, and 25% not able to work all of the time because of the condition. The findings of this survey are corroborated by others [6].

The most significant result of this survey is the insight into patients' journey to diagnosis. At the time of this survey, patients had been experiencing fibromyalgia symptoms for an average of 6.5 years. Based on their own recollection, patients experienced symptoms for an average of 11 months before presenting to a physician. Care should be taken when interpreting data on the time lag between first symptoms and presentation to a physician, as patients may have a history of short and transient episodes of pain and other symptoms before the development of persistent symptoms. Some patients seem to have a very precise time of onset of symptoms e.g. following a traumatic event, whereas others may have a long history of localized pain or other symptoms (personal observations). The most common reason (74%) for waiting to present to the doctor was the belief that symptoms would resolve. Given that fibromyalgia symptoms may appear but not persist throughout the day and that symptoms may fluctuate in severity, it is not surprising that patients do wait some time before seeking help from a physician. Half of the patients also waited to present to a physician, because they did not like taking medications or did not like going to the doctor, and 68% of patients thought they could manage symptoms themselves. Based on clinical experience, patients seek help from physicians not because of their symptoms *per se*, but because they have a poor quality of life that is a consequence of their fibromyalgia symptoms and are seeking help to improve quality of life. Patients may struggle for some time with impaired quality of life before seeking help.

From the time patients recalled first presenting to the doctor for their fibromyalgia symptoms, it took on average 2.3 years and presenting to 3.7 physicians before receiving a diagnosis. Such a delay can contribute to patient frustration, as White et al. showed that a diagnosis of fibromyalgia improves satisfaction [5]. Furthermore, this survey extends the result of previous research in the UK showing that patients with fibromyalgia are often referred to multiple specialists and have numerous investigations before the diagnosis is established [9]. This survey shows that delayed diagnosis is not unique for the UK but also occurs across other countries and cultures. Importantly, the previous UK study showed that the diagnosis of fibromyalgia reduced healthcare utilization including referrals and investigations [9]. Hence educating clinicians on the recognition and diagnosis of fibromyalgia would benefit both patients and healthcare providers. Therefore, it is important to understand why the diagnosis of fibromyalgia is delayed. Several potential factors are revealed by this survey.

First, over half the patients in this survey found it difficult to communicate with their physician, and most felt that doctors needed to focus more on symptoms and spend more time with patients to reach a diagnosis. Therefore, multiple symptoms compounded by limited consultation time may be an important factor. Second, many physicians are not aware of the ACR criteria. In a small percentage of those who are aware of the ACR criteria and have diagnosed fibromyalgia patients in the past two years, the ACR criteria are not used in routine clinical practice. Better medical education to improve knowledge and application of the ACR criteria may reduce delay in the diagnosis of fibromyalgia. Third, ACR criteria recognize pain and tender points but ignore the other fibromyalgia symptoms. This could lead to confusion. Improving the diagnostic criteria may help to circumvent this problem.

An important limitation of all opinion research, and this study is not an exception, is that respondents may not perfectly well recall their experiences and feelings. Respondents' feelings, attitudes and perceptions are subject to some potential changes in the course of time. The survey provides a snapshot of the respondents' experiences and does not seek to address how these might have changed longitudinally. We sought to ensure that the diagnosis of fibromyalgia was not entirely dependent on the patients' recollection, by including patients who were diagnosed with fibromyalgia by a physician. The main reason patients were recruited through their physicians, rather than by random sampling as is sometimes the case with such research, was to ensure that only patients who were indeed diagnosed with fibromyalgia by their physicians were interviewed.

## Conclusion

This survey of 800 patients with fibromyalgia from eight countries provides further evidence that fibromyalgia is characterized by multiple symptoms, including pain, with a notable impact on quality of life and function. Current treatment regimens are not satisfactory for a substantial proportion of patents. Patients may experience symptoms for some time before seeking the help of physicians. Patient experiences suggest that physicians find diagnosis difficult and that physicians need to spend more time with patients to diagnose fibromyalgia. Patients who reported dissatisfaction with treatment also reported waiting longer to receive a work-up, diagnosis and care for their fibromyalgia. By the time the patient presents to the physician who actually diagnosed their condition, the fibromyalgia journey has already begun (on average over 2 years). The association between chronicity of symptoms and worse response to treatment suggests that earlier diagnosis and treatment may lead to improved treatment response and reduced impact of the condition.

## Competing interests

EC has received fees for consulting to Pfizer. SP has received fees for consulting to Pfizer. JK was an employee of Pfizer Inc at the time the survey was conducted.

TL is an employee of Pfizer Inc. AG and EK are employed by Harris Interactive who was commissioned and funded by Pfizer Inc to develop and conduct the survey.

## Authors' contributions

All authors gave final approval of the version submitted for publication.

EC and SP made substantial contributions to the interpretation of data and were involved in reviewing and critiquing the manuscript for important intellectual content. TL and JK made substantial contributions to the conception and design of the survey and interpretation of data and were involved in reviewing and critiquing the manuscript for important intellectual content. AG made substantial contributions to conception and design, acquisition of data, and analysis and interpretation of data and was involved in reviewing and critiquing the manuscript for important intellectual content. EK made substantial contributions to the conception and design of the survey, interpretation of data and was involved in reviewing and critiquing the manuscript for important intellectual content.

## Authors' information

Profs Perrot and Choy are both rheumatologists who have an interest in fibromyalgia and are involved with management of patients, clinical research, as well as medical education.

## Pre-publication history

The pre-publication history for this paper can be accessed here:

http://www.biomedcentral.com/1472-6963/10/102/prepub

## Supplementary Material

Additional file 1 **Survey of patients with fibromyalgia**. This is a PDF of the patient survey. Click here for file

## References

[B1] White KP Harth M Classification, epidemiology, and natural history of fibromyalgia Curr Pain Headache Rep 2001 5 4 320 9 10.1007/s11916-001-0021-2 11403735

[B2] Wolfe F Ross K Anderson J Russell IJ Hebert L The prevalence and characteristics of fibromyalgia in the general population Arthritis Rheum 1995 38 1 19 28 10.1002/art.1780380104 7818567

[B3] Wolfe F Smythe HA Yunus MB Bennett RM Bombardier C Goldenberg DL Tugwell P Campbell SM Abeles M Clark P Fam AG Farber SJ Fiechtner JJ Franklin CM Gatter RA Hamaty D Lessard J Lichtbroun AS Masi AT McCain GA Reynolds WJ Romano TJ Russell IJ Sheon RP The American College of Rheumatology 1990 criteria for the classification of fibromyalgia. Report of the multicenter criteria committee Arthritis Rheum 1990 33 2 160 172 10.1002/art.1780330203 2306288

[B4] Mease P Fibromyalgia syndrome: review of clinical presentation, pathogenesis, outcome measures, and treatment J Rheumatol 2005 32 Suppl 75 6 21 16078356

[B5] White KP Nielson WR Harth M Ostbye T Speechley M Chronic widespread musculoskeletal pain with or without fibromyalgia: psychological distress in a representative community adult sample J Rheumatol 2002 29 3 588 94 11908578

[B6] Arnold LM Crofford LJ Mease PJ Burgess SM Palmer SC Abetz L Martin SA Patient perspectives on the impact of fibromyalgia Patient Educ Couns 2008 73 1 114 20 10.1016/j.pec.2008.06.005 18640807 PMC2564867

[B7] Hoffman DL Dukes EM The health status burden of people with fibromyalgia: a review of studies that assessed health status with the SF-36 or the SF-12 Int J Clin Pract 2008 62 1 115 26 10.1111/j.1742-1241.2007.01638.x 18039330 PMC2228371

[B8] Perrot S Fibromyalgia syndrome: a relevant recent construction of an ancient condition? Curr Opin Support Palliat Care 2008 2 2 122 7 10.1097/SPC.0b013e3283005479 18685409

[B9] Hughes G Martinez C Myon E Taïeb C Wessely S The impact of a diagnosis of fibromyalgia on health care resource use by primary care patients in the UK: an observational study based on clinical practice Arthritis Rheum 2006 54 1 177 83 10.1002/art.21545 16385513

[B10] Bennett RM Jones J Turk DC Russell IJ Matallana L An internet survey of 2,596 people with fibromyalgia BMC Musculoskelet Disord 2007 8 27 10.1186/1471-2474-8-27 17349056 PMC1829161

[B11] Mease P Arnold LM Bennett R Boonen A Buskila D Carville S Chappell A Choy E Clauw D Dadabhoy D Gendreau M Goldenberg D Littlejohn G Martin S Perera P Russell IJ Simon L Spaeth M Williams D Crofford L Fibromyalgia syndrome J Rheumatol 2007 34 6 1415 25 17552068

[B12] National Fibromyalgia Research Association Web site http://www.nfra.net/Diagnost.htm Accessed 23 January 2009

