# Supplementary material for: A patient survey of the impact of fibromyalgia and the journey to diagnosis
Source: BMC Health Serv Res. 2010 Apr 26;10:102. doi: 10.1186/1472-6963-10-102 (PMC2874550; doi:10.1186/1472-6963-10-102)
Supplement: Additional file 1 — Survey of patients with fibromyalgia. This is a PDF of the patient survey. [file 1472-6963-10-102-S1.PDF]

HARRIS INTERACTIVE
5 Independence Way
Princeton, NJ 08540

Project Manager/Researcher: Anna Ginovker
Email: aginovker@harrisinteractive.com
Phone: 609-919-2638

Project Controller: Sarah Schreiber
Email: sschreiber@harrisinteractive.com
Phone: 404-228-8933

J31666B
1/17/08

Fibromyalgia Global Study - PATIENT Survey

CLIENT: Pfizer

Information for Online Surveys

Title for initial survey page (Required for online jobs): Health Survey

Number of Response Equivalents (REs): XX

Wave Number/Version/Languages (if applicable): British English, French, German, Italian, Spanish, Dutch, Mexican Spanish, Korean

Demographics Template (Required for online jobs): Custom

SECTION 400: SCREENING QUESTIONS

BASE: ALL RESPONDENTS
Q404 Thank you very much for calling us! I'm ________ from Harris Interactive, a global market research firm. We invite you to participate in this telephone survey about pain to tell us about some of your experiences and opinions. Your participation will help make this important study a success.

First, may I have the ID number that your physician gave you?

[PN: 10-DIGIT NUMERIC; AT LEAST 8-DIGITS MANDATORY]

RECORD # I_I_I_I_I_I_I_I_I_I_I

BASE: ALL RESPONDENTS
Q410 Which of the following categories best describes your age?
(INTERVIEWER: IF RESPONDENT IS NOT SURE OR DECLINES TO ANSWER, PROBE TO CONFIRM RESPONDENT IS AT LEAST 18 YEARS OF AGE OR OLDER. GENTLY PROBE FOR APPROXIMATE AGE RANGE. UPDATE RESPONSE CHOICE AS NECESSARY. RESPONDENT MUST CONTINUE EVEN IF THE AGE RANGE IS NOT IDENTIFIED UNLESS THERE IS A STRONG ENOUGH REASON TO BELIEVE THAT RESPONDENT IS UNDER THE AGE OF 18.)

1. Under 18 [TERMINATE AND SKIP TO Q480]
2. 18-35
3. 36-44
4. 45-59
5. 60-74
6. 75 and over
8. Not sure (V)
9. Decline to answer (V)

BASE: ALL RESPONDENTS AGED 18+ (Q410/2-9)
Q412 GENDER (INTERVIEWER: RECORD GENDER. DO NOT ASK)
1. MALE
2. FEMALE

BASE: RESIDENT OF UK, GERMANY, FRANCE, ITALY, SPAIN, NETHERLANDS, MEXICO OR S. KOREA AND 18+ (Q455/1-8 AND Q410/2-9)
Q435 Has a physician diagnosed you with fibromyalgia?

1. Yes
2. No TERMINATE
8. Not sure (V) TERMINATE
9. Decline to answer (V) TERMINATE

BASE: ALL RESPONDENTS
Q480 – INITIAL CLASSIFICATION QUESTION – BEHIND THE SCENES

PROGRAMMER NOTE:
GET CODE 1 IF:
- 18 years or older, OR Not sure, OR Decline to answer (Q410/2-9) AND
- Reside in UK, France, Germany, Spain, Italy, Netherlands, Mexico or S. Korea (Q455/1-8) AND
- Diagnosed with fibromyalgia (Q435/1)

ALL OTHERS GET CODE 2

1. Qualified
2. Not Qualified

BASE: ALL RESPONDENTS
Q485 – COUNTRY QUOTA QUESTION – BEHIND THE SCENES

GET CODE 1 IF Q455/1
GET CODE 2 IF Q455/2
GET CODE 3 IF Q455/3
GET CODE 4 IF Q455/4
GET CODE 5 IF Q455/5
GET CODE 6 IF Q455/6
GET CODE 7 IF Q455/7
GET CODE 8 IF Q455/8

1.	UK	N=100
2.	FRANCE	N=100
3.	GERMANY	N=100
4.	ITALY	N=100
5.	SPAIN	N=100
6.	NETHERLANDS	N=100
7.	MEXICO	N=100
8.	SOUTH KOREA	N=100

BASE: ALL RESPONDENTS
Q486 - COUNTRY QUOTA CHECK QUESTION
[PROGRAMMER NOTE: CHECK QUOTA AT Q485.]

1 QUOTA MET
2 QUOTA NOT MET
3 QUOTA NOT FOUND

BASE: ALL RESPONDENTS
Q487 - QUOTA BY AGE AND COUNTRY QUOTA QUESTION

GET CODE 1 IF (Q455/1 AND Q412/2 AND Q480/1)
GET CODE 2 IF (Q455/1 AND Q412/1 AND Q480/1)
GET CODE 3 IF (Q455/2 AND Q412/2 AND Q480/1)
GET CODE 4 IF (Q455/2 AND Q412/1 AND Q480/1)
GET CODE 5 IF (Q455/3 AND Q412/2 AND Q480/1)
GET CODE 6 IF (Q455/3 AND Q412/1 AND Q480/1)
GET CODE 7 IF (Q455/4 AND Q412/2 AND Q480/1)
GET CODE 8 IF (Q455/4 AND Q412/1 AND Q480/1)
GET CODE 9 IF (Q455/5 AND Q412/2 AND Q480/1)
GET CODE 10 IF (Q455/5 AND Q412/1 AND Q480/1)
GET CODE 11 IF (Q455/6 AND Q412/2 AND Q480/1)
GET CODE 12 IF (Q455/6 AND Q412/1 AND Q480/1)
GET CODE 13 IF (Q455/7 AND Q412/2 AND Q480/1)
GET CODE 14 IF (Q455/7 AND Q412/1 AND Q480/1)
GET CODE 15 IF (Q455/8 AND Q412/2 AND Q480/1)

GET CODE 16 IF (Q455/5 AND Q412/1 AND Q480/1)

1. UK WOMEN N=999
2. UK MEN N=999
3. FRANCE WOMEN N=999
4. FRANCE MEN N=999
5. GERMANY WOMEN N=999
6. GERMANY MEN N=999
7. ITALY WOMEN N=999
8. ITALY MEN N=999
9. SPAIN WOMEN N=999
10. SPAIN MEN N=999
11. NETHERLANDS WOMEN N=999
12. NETHERLANDS MEN N=999
13. MEXICO WOMEN N=999
14. MEXICO MEN N=999
15. S. KOREA WOMEN N=999
16. S. KOREA MEN N=999

BASE: ALL RESPONDENTS
Q489 - QUOTA BY AGE AND COUNTRY QUOTA CHECK QUESTION
[PROGRAMMER NOTE: CHECK QUOTA AT Q487.]

1 QUOTA MET
2 QUOTA NOT MET
3 QUOTA NOT FOUND

BASE: ALL RESPONDENTS
Q99 SCREENER QUALIFICATION IDENTIFICATION QUESTION (DOES NOT APPEAR ON SCREEN)

[PN: GET CODE 1 IF Q480/1 AND Q485/1-8 AND Q486/2, 3 AND Q487/1-16 AND Q489/2, 3. GET CODE 3 IF Q480/1 AND Q485/1-8 AND Q486/1 AND Q487/1-16 AND Q489/1. ALL OTHERS GET CODE 6.

1 SCREENER QUALIFIED RESPONDENTS, QUOTA OPEN
3 SCREENER QUALIFIED RESPONDENTS, QUOTA CLOSED
6 NOT SCREENER QUALIFIED

SECTION 500: MAIN QUESTIONNAIRE—FIBROMYALGIA SYMPTOMS

Now, I am going to ask you some questions about your fibromyalgia symptoms.

BASE: ALL QUALIFIED RESPONDENTS (Q99/1)
Q500 Please indicate which of the following symptoms of fibromyalgia you have experienced. (INTERVIEWER READ LIST. ALLOW MULTIPLE RESPONSES.)

[RANDOMIZE]
1. Chronic widespread pain
2. Problems sleeping
3. Fatigue
4. Headaches
5. Facial pain
6. Heightened sensitivity to touch
7. Difficulty concentrating
8. Numbness and/or tingling sensations
9. Feelings of anxiety
10. Feelings of depression
11. Joint pain
12. Stiffness
13. Leg cramps
14. Low back pain
96. Other [ANCHOR]
97. None of these [ANCHOR, E]
98. Not sure (V, E)
99. Decline to answer (V, E)

BASE: QUALIFIED RESPONDENTS WHO MENTIONED AT LEAST ONE LISTED SYMPTOM (Q500/1-14,96)

Q510 Please tell me how disruptive each of these symptoms is to the overall quality of your life. Would you say it is not at all disruptive, not very disruptive, fairly disruptive, very disruptive, or extremely disruptive? (INTERVIEWER: READ LIST AND REPEAT SCALE IF NECESSARY.)

[PROGRAMMER: DISPLAY ONLY SYMPTOMS THAT RESPONDENT MENTIONED IN Q500.]

Q511

1	2	3	4	5	8	9
Not at all disruptive	Not very disruptive	Fairly disruptive	Very disruptive	Extremely disruptive	Not Sure (V)	Decline to Answer (V)

[RANDOMIZE]

1. Chronic widespread pain
2. Problems sleeping
3. Fatigue
4. Headaches
5. Facial pain
6. Heightened sensitivity to touch
7. Difficulty concentrating
8. Numbness and/or tingling sensations
9. Feelings of anxiety
10. Feelings of depression
11. Joint pain
12. Stiffness
13. Leg cramps
14. Low back pain
96. Other

BASE: RESPONDENTS WHO HAVE EXPERIENCED CHRONIC WIDESPREAD PAIN (Q500/1)

Q515 In a typical week, how often would you say you experience chronic widespread pain from fibromyalgia? Would you say…? (INTERVIEWER: READ LIST)

1. Less than once a week
2. Once a week
3. 2 to 3 times per week
4. 4 to 5 times per week
5. Every day
8. Not sure (V)
9. Decline to answer (V)

BASE: RESPONDENTS WHO HAVE EXPERIENCED CHRONIC WIDESPREAD PAIN (Q500/1)

Q520 In general, how severe is the chronic widespread pain you experience from your fibromyalgia? Please use a scale from 0-10, where '0' means no pain and '10' means the worst pain imaginable.

1. 0 – No pain
2. 1
3. 2
4. 3
5. 4
6. 5
7. 6
8. 7
9. 8
10. 9
11. 10 – Worst pain

98 Not sure (V)
99 Decline to answer (V)

BASE: ALL QUALIFIED RESPONDENTS (Q99/1)

Q525 How long ago did you first notice the symptoms of fibromyalgia?

(INTERVIEWER: RECORD LENGTH OF TIME AS INDICATED BY RESPONDENT.

- IF RESPONDENT PROVIDES RESPONSE THAT IS LESS THAN ONE MONTH, I.E 3 WEEKS, RECORD AS ONE MONTH.)

l_l_l year(s) (Example: 03)
l_l_l month(s) (Example: 10)
[YEARS AND MONTHS MANDATORY (DOUBLE DIGIT)]
[RANGE: 00-95]

98 – Not Sure (V, E)
99 – Decline to Answer (V, E)

BASE: ALL QUALIFIED RESPONDENTS (Q99/1)

Q530 How long did it take you to see a physician after you first experienced your fibromyalgia symptoms?

(INTERVIEWER: RECORD LENGTH OF TIME AS INDICATED BY RESPONDENT.

- IF RESPONDENT PROVIDES RESPONSE THAT IS LESS THAN ONE WEEK, I.E 5 DAYS, RECORD AS ONE WEEK.)

l_l_l year(s) (Example: 03)
l_l_l month(s) (Example: 10)
l_l_l week(s) (Example: 01)
[YEARS, MONTHS, AND WEEKS MANDATORY (DOUBLE DIGIT)]
[RANGE: 00-95]

98 Not Sure (V, E);
99 Decline to Answer (V, E)

BASE: WAITED 4 WEEKS OR LONGER (Q537/4 WEEKS OR MORE)

Q535 I am going to read you a list of reasons why some people wait before seeing a physician about their symptoms. For each reason, please tell me whether or not it was a reason you waited before seeing a physician. (INTERVIEWER: READ EACH STATEMENT FROM THE LIST BELOW AND AFTER EACH STATEMENT SAY: 'Was it a reason?')

Yes	No	Not Sure	Decline to Answer
1	2	8	9

[RANDOMIZE]

1. The symptoms were not severe enough
2. You were afraid that your physician would not take you seriously
3. You thought the symptom(s) might go away by themselves
4. You do not like going to a physician
5. You do not like receiving treatments like medications or injections
6. You were concerned about the cost of a physician visit or treatment
7. You were too busy
8. It was difficult to schedule an appointment with the physician
9. You thought you could manage the symptoms yourself

SECTION 600: MAIN QUESTIONNAIRE—FIBROMYALGIA DIAGNOSIS/ATTITUDES TOWARD FIBROMYALGIA

Now I am going to ask you some questions about your fibromyalgia diagnosis.

BASE: ALL QUALIFIED RESPONDENTS (Q99/1)

Q600 From the time you first saw a physician about the symptoms you were experiencing, how long did it take before you were diagnosed with fibromyalgia?

(INTERVIEWER: RECORD LENGTH OF TIME AS INDICATED BY RESPONDENT.

- IF RESPONDENT PROVIDES RESPONSE THAT IS LESS THAN ONE WEEK, I.E 5 DAYS, RECORD AS ONE WEEK.)

l_l_l year(s) (Example: 03)
l_l_l month(s) (Example: 10)
l_l_l week(s) (Example: 01)
[YEARS, MONTHS, AND WEEKS MANDATORY (DOUBLE DIGIT)]
[RANGE: 00-95]

____________97- Diagnosed on first visit to a physician about my symptoms (V, E)
98 – Not Sure (V, E)
99 – Decline to Answer (V, E)

[PN: IF Q600/NE 97 CONTINUE WITH Q605. IF Q600/97 SKIP TO Q610.]

BASE: NOT DIAGNOSED ON FIRST VISIT (Q600/NE 97)

Q605 All together**,** how many physicians did you see to receive your fibromyalgia diagnosis?

(INTERVIEWER: ENTER 98 FOR NOT SURE, 99 FOR DECLINE TO ANSWER)

l_l_l physicians [RANGE: 1-95; 98, 99]

BASE: ALL QUALIFIED RESPONDENTS (Q99/1)

Q610 From the time you first saw a physician about your symptoms and up until now, what type(s) of physicians did you see about your symptoms of fibromyalgia? **(**INTERVIEWER: READ LIST. ALLOW MULTIPLE RESPONSES.)

1. General Practitioner/Family Practitioner
2. Internist/Internal Medicine
3. OBGYN
4. Rheumatologist
5. Neurologist
6. Psychiatrist
7. Pain Specialist
8. Orthopedist
9. Physical Therapist
10. Anesthesiologist
96. Other
98. Not sure (V, E)
99. Decline to answer (V, E)

BASE: ALL QUALIFIED RESPONDENTS (Q99/1)

Q616 What kind of physician diagnosed you with fibromyalgia? (INTERVIEWER: READ LIST IF NECESSARY. SINGLE RESPONSE.)

1. General Practitioner/Family Practitioner
2. Internist/Internal Medicine
3. OBGYN
4. Rheumatologist
5. Neurologist
6. Psychiatrist
7. Pain Specialist
8. Orthopedist
9. Physical Therapist
10. Anesthesiologist
96. Other
98. Not sure (V, E)
99. Decline to answer (V, E)

BASE: ALL QUALIFIED RESPONDENTS (Q99/1)

Q623 What kind of physician is currently treating your fibromyalgia? (INTERVIEWER: READ LIST IF NECESSARY. MULTIPLE RESPONSE.)

1. General Practitioner/Family Practitioner
2. Internal Medicine/Internist
3. OBGYN
4. Rheumatologist
5. Neurologist
6. Psychiatrist
7. Pain Specialist
8. Orthopedist
9. Physical Therapist
10. Anesthesiologist
96. Other
98. Not sure (V, E)
99. Decline to answer (V, E)

BASE: ALL QUALIFIED RESPONDENTS (Q99/1)

Q625 Overall, how easy or how difficult was your experience in receiving a fibromyalgia diagnosis? Would you say…? (INTERVIEWER: READ LIST)

1. Very easy
2. Somewhat easy
3. Neither easy nor difficult
4. Somewhat difficult
5. Very difficult
8. Not sure (v)
9. Decline to answer (v)

BASE: ALL QUALIFIED RESPONDENTS (Q99/1)

Q640 Now, I am going to read you several general statements about fibromyalgia. Based on your personal experience being diagnosed and treated for fibromyalgia, please indicate if you strongly agree, somewhat agree, neither agree nor disagree, somewhat disagree or strongly disagree with each of the following statements. (INTERVIEWER: READ LIST. REPEAT SCALE IF NECESSARY.)

Q641

Strongly Agree	Somewhat Agree	Neither Agree Nor Disagree	Somewhat Disagree	Strongly Disagree	Not Sure (V)	Decline to Answer (V)
1	2	3	4	5	8	9

[RANDOMIZE]

1. Physicians are well trained to diagnose and treat fibromyalgia
2. Physicians are compassionate with their fibromyalgia patients
3. Fibromyalgia patients have had at least one experience where a physician did not take them seriously
4. Physicians need to spend more time with patients to identify fibromyalgia
5. Physicians think that fibromyalgia patients exaggerate their symptoms
6. Fibromyalgia patients find it difficult to communicate their symptoms to physicians
7. Physicians need to focus more on fibromyalgia symptoms

BASE: ALL QUALIFIED RESPONDENTS (Q99/1)

Q645 Thinking about all of the physicians you have seen about your fibromyalgia symptoms, overall, how caring do you feel they have been towards you? Would you say…?

1. Extremely caring
2. Very caring
3. Fairly caring
4. Not very caring
5. Not at all caring
8. Not sure (V)
9. Decline to answer (V)

BASE: ALL QUALIFIED RESPONDENTS (Q99/1)

Q650 In a typical month, how often do you visit a physician about your fibromyalgia symptoms? Would you say…?

1. Less than once a month
2. Once a month
3. Twice a month
4. Three times per month or more
8. Not sure (V)
9. Decline to answer (V)

SECTION 700: MAIN QUESTIONNAIRE— IMPACT ON QUALITY OF LIFE

BASE: ALL QUALIFIED RESPONDENTS (Q99/1)

Q705 Now I am going to read out a list of some aspects of your life that may have been impacted by fibromyalgia. For each aspect, please tell me if fibromyalgia has had a very strong impact, strong impact, moderate impact, slight impact or no impact?

(INTERVIEWER: READ OUT LIST AND REPEAT SCALE IF NECESSARY.)

Q706

Very Strong Impact	Strong Impact	Moderate Impact	Slight Impact	No Impact	Not Sure (V)	Decline to Answer (V)
1	2	3	4	5	8	9

[RANDOMIZE]

1. Overall quality of your life [ANCHOR]
2. Your personal relationships
3. Your ability to keep commitments or appointments
4. Your ability to participate in hobbies
5. Your ability to care for family members and children
6. Your sex life
7. Your physical mobility
8. Your overall mood
9. Your concentration or memory
10. Your motivation or drive

BASE: ALL QUALIFIED RESPONDENTS (Q99/1)

Q715 If you had to estimate, how many days of work did you miss over the past 12 months because of your fibromyalgia? (INTERVIEWER: READ OUT LIST.)

1. None – you have not missed any days
2. 1 to 9 days
3. 10 to 20 days
4. 21 to 40 days
5. More than 40 days
6. You have not been employed in the past 12 months
8. Not sure (V)
9. Decline to answer (V)

BASE: ALL QUALIFIED RESPONDENTS (Q99/1)

Q726 Please tell me whether each of the following statements applies to you? (INTERVIEWER: READ LIST.)

Q727

Applies to You	Doesn't Apply to You	Not Sure (V)	Decline to Answer (V)
1	2	8	9

[RANDOMIZE]

1. Due to your fibromyalgia you are not able to work and do not earn an income
2. Due to your fibromyalgia you can only work sometimes and do not earn as much as you used to earn
3. You lost your job due to your fibromyalgia
4. You have spent a lot of money out-of-pocket on medical care due to your fibromyalgia
5. You have been held back in your career due to your fibromyalgia
6. You have paid out-of-pocket for assistance in performing daily activities due to your fibromyalgia

BASE: ALL QUALIFIED RESPONDENTS (Q99/1)

Q731 Now I would like to ask you about the level of understanding your family and friends have expressed towards your condition. Would you say your family and friends have been extremely understanding, very understanding, fairly understanding, not very understanding or not at all understanding about the following? (INTERVIEWER: READ OUT LIST. REPEAT SCALE IF NECESSARY.)

Q732

Extremely Understanding	Very Understanding	Fairly Understanding	Not Very Understanding	Not At All Understanding	Not Sure (V)	Decline to Answer (V)
1	2	3	4	5	8	9

[RANDOMIZE]

1. Your fibromyalgia overall [ANCHOR]
2. You staying home from a family outing due to your fibromyalgia
3. You taking a sick day at work due to your fibromyalgia
4. You needing help with daily chores due to your fibromyalgia
5. You needing to discuss your fibromyalgia with your family and friends

SECTION 800: MAIN QUESTIONNAIRE— FIBROMYALGIA TREATMENT

Now I'm going to ask you some questions about how your fibromyalgia is treated.

BASE: ALL QUALIFIED RESPONDENTS (Q99/1)

Q800 What treatments are you currently using to treat your fibromyalgia? (INTERVIEWER READ LIST. ALLOW MULTIPLE RESPONSES.)

[RANDOMIZE]

1. Pain reliever(s) you can buy over-the-counter, without a prescription
2. Pain reliever(s) prescribed by your physician
3. Other drug(s) prescribed by your physician
4. Sleep aids
5. Counseling with a mental health professional
6. Relaxation techniques
7. Biofeedback
8. Lifestyle changes
9. Exercise

96. Other [ANCHOR]

97 None (V) [ANCHOR, E]

98 Not sure (V)[ANCHOR, E]

99 Decline to answer (V) [ANCHOR, E]

[PN: IF Q800/1-9, 96 CONTINUE WITH Q805. IF Q800/97, 98, 99 SKIP TO Q900.]

BASE: CURRENTLY USING AT LEAST ONE OF THE LISTED TREATMENTS (Q800/1-9, 96)

Q805 How satisfied are you with your current overall treatment's ability to relieve your fibromyalgia symptoms? Would you say…? (INTERVIEWER: READ LIST.)

1. Extremely satisfied
2. Very satisfied
3. Fairly satisfied
4. Not very satisfied
5. Not at all satisfied

8. Not sure (V)
9. Decline to answer (V)

BASE: QUALIFIED RESPONDENTS WHO MENTIONED AT LEAST ONE LISTED SYMPTOM AND CURRENTLY USE AT LEAST ONE OF THE LISTED TREATMENTS (Q500/1-14,96 AND Q800/1-9, 96)

Q810 Of the fibromyalgia symptoms that you have experienced, which ones, if any, do you feel are not well managed by your current treatment? (INTERVIEWER: READ LIST. ALLOW MULTIPLE RESPONSES.)

[PN: PROGRAMMER: DISPLAY ONLY SYMPTOMS THAT RESPONDENT MENTIONED IN Q500.]

[RANDOMIZE]

1. Chronic widespread pain
2. Problems sleeping
3. Fatigue
4. Headaches
5. Facial pain
6. Heightened sensitivity to touch
7. Difficulty concentrating
8. Numbness and/or tingling sensations
9. Feelings of anxiety
10. Feelings of depression
11. Joint pain
12. Stiffness
13. Leg cramps
14. Low back pain
96. Other [ANCHOR]
97. None (V) [ANCHOR, E]
98. Not sure (V) [ANCHOR, E]
99. Decline to answer (V) [ANCHOR, E]

SECTION1000: THANK YOU

BASE: QUALIFIED PATIENTS (Q99/1)
Q299 Those are all the questions we have for you. Thank you very much for participating in this survey. Have a great day.

BASE: ALL RESPONDENTS
Q59 STATUS OF RESPONDENT (LABELS USED IN ICW SAMPLE DISPOSITION REPORTS)

29 OVER QUOTA (Q99/3)
41 SCREENER REFUSAL #1 (TBD)
44 SCREENER REFUSAL #4 (Q435/9)
61 NOT QUALIFIED #1 (Q410/1)
63 NOT QUALIFIED #3 (Q435/2,8)
999 COMPLETE (Q99/1)

BASE: ALL RESPONDENTS
Q60 STATUS OF RESPONDENT (DOES NOT APPEAR ON SCREEN)

[PN: GET CODES AT Q99]

1 QUALIFIED RESPONDENTS, QUOTA OPEN
2 PARTIALLY QUALIFIED, QUOTA OPEN
3 QUALIFIED RESPONDENTS, QUOTA CLOSED
4 PARTIALLY QUALIFIED RESPONDENTS, QUOTA CLOSED
5 OVERALL QUOTA CLOSED
6 NOT QUALIFIED
